# The London Ragamuffin

**Published:** 1885-07

**Authors:** 


### The London Ragamuffin.

The genuine ragamuffin will never complain. He never expects or even hopes that his condition will improve; he is as much a fatalist as the Turk. I once asked an interesting little boy with a pale, careworn face and an intelligent expression, if he had ever wondered why it was that he had nothing but rags; why it was he had no boots, and sometimes no bread to eat, while I had plenty of everything? He looked up at me with a calm, patient expression, as much as to say, "I have never wondered at such things." "Tell me," I persisted, "have you ever thought about this difference?" "It's the Lord's will," he replied tritely; but he seemed reluctant, when I pressed him, to explain what he understood by the Lord's will. At last in a timid, hurried voice he said: "It is all the Lord's doing, this way: you are grand like, and dress nice, and lives in a big house, and you have a pianner, and—and," he looked around the room that he might enumerate all our titles to consideration—"and a sofy: so the Lord sees as how you are gentlefolks, and He thinks lots of such like as you. But we are very poor, we are. Mother pawns the blankets, and father beats mother, and swears awful. We ain't got no Sunday things ; we're all raggety, so the Lord don't take much notice on us."—*The English Illustrated Magazine.*

